# Control effects and mechanisms of metabolites from *Streptomyces ahygroscopicus* var. gongzhulingensis strain 769 on sclerotinia rot in sunflowers

**DOI:** 10.3389/fpls.2025.1649819

**Published:** 2025-09-11

**Authors:** Zhiming Liu, Yang Lu, Qiyun Li, Zhao Xie, Li Sui, Shufang Gong, Zhengkun Zhang

**Affiliations:** ^1^ College of Horticulture and Landscape Architecture, Northeast Agricultural University, Harbin, China; ^2^ Institute of Plant Protection, Jilin Academy of Agricultural Sciences, Jilin Key Laboratory of Agricultural Microbiology, Gongzhuling, China; ^3^ Key Laboratory of Integrated Pest Management on Crops in Northeast China, Ministry of Agriculture and Rural Areas, Gongzhuling, China; ^4^ Jilin Agricultural University, Changchun, China

**Keywords:** Streptomyces, *Sclerotinia sclerotiorum*, *Helianthus annuus*, biocontrol, microbiomics

## Abstract

**Introduction‌:**

Sunflower sclerotinia rot caused by *Sclerotinia sclerotiorum* poses a significant threat to global agriculture. This study investigates Streptomyces ahygroscopicus var. gongzhulingensis 769 (S769) as a novel biocontrol agent against this devastating disease.

**Methods‌‌:**

Antagonism assays evaluated S769's efficacy in vitro, while detached leaf, pot, and field trials assessed disease suppression through soil mixing (S769-Ms) and root drenching (S769-i). Mechanistic analyses included enzyme activity assays, microbiome profiling, qPCR quantification of pathogen load, and root transcriptomics.

**Results‌‌:**

S769 exhibited 65.79% mycelial growth inhibition in vitro. Field trials demonstrated significant disease control (S769-Ms: 7.36%, S769-i: 5.92% vs. 14.69% control), with 15.85% increase in root fresh weight and 34.26% reduction in shriveled seeds. qPCR confirmed 4.85-fold pathogen reduction in leaves and 2.68-fold in roots. Transcriptomics revealed 6,622 upregulated genes, including MAPK signaling and phenylpropanoid biosynthesis pathways.

**Discussion‌‌:**

S769 demonstrates dual action through direct antifungal effects and host defense activation. The enrichment of beneficial rhizobacteria (Sphingomonas, Chitinophagaceae) without altering microbial diversity highlights its potential as a sustainable agricultural solution for sclerotinia rot management.

## Highlights

- It was observed that *S*. *ahygroscopicus* var. gongzhulingensis 769 effectively inhibits the growth of *Sclerotinia sclerotiorum*, the pathogen responsible for sunflower sclerotinia stem rot.
- Treatment with metabolites from *S*. *ahygroscopicus* var. gongzhulingensis 769 (S769) significantly reduced disease severity on sunflower leaves and decreased the infection rate of sclerotinia stem rot, while also promoting plant growth.
- Field trials demonstrated that sunflower roots treated with S769 exhibited improved health, resulting in plumper seeds.
- S769 induces an increase in plant defense enzyme activity, enhances soil enzyme activity, and contributes positively to both soil health and plant stress resistance.

## 1 Introduction

Sunflower sclerotinia rot, also known as white rot, is a serious fungal disease caused by *Sclerotinia sclerotiorum*, which is a widely distributed soil born phytopathogen (da Silva et al., 2022). It infects a range of crops including soybeans, sunflowers (*Helianthus annuus*), and rapeseed, leading to sclerotinia diseases in these hosts. Sclerotinia rot is particularly prevalent and challenging to manage in sunflower production, posing a significant threat to crop yields (Harveson et al., 2016). Infected plants often show symptoms such as stem rot and wilting. In severe cases, they may fall over and die (Markell et al., 2023). Moreover, sclerotia can survive in the soil for several years and are difficult to eradicate (Liu et al., 2017). In addition, the ascospores produced by germinated sclerotia can cause sunflower head rot, increasing the number of shriveled seeds and even leading to total crop failure (Markell et al., 2023). Currently, sunflower sclerotinia disease has become one of the most significant factors affecting sunflower yield and quality. According to statistics, in the United States, the annual sunflower yield loss caused by *Sclerotinia* infection accounts for approximately 10–20% of the total yield (Gulya et al., 2019). Moreover, in 1988, in Buenos Aires Province, Argentina, sunflower head rot caused by sclerotinia disease led to a 100% reduction in sunflower production, resulting in severe economic losses (Liu et al., 2018).

Chemical control remains the predominant approach for managing sclerotinia disease. Although it has achieved certain control effects, the continuous application of such chemicals has led to the emergence of the “3R” problem, namely pesticide residues, resurgence of pests and diseases, and development of resistance in target organisms (Sparks and Bryant, 2022). Excessive use of chemical pesticides not only aggravates the resistance of *Sclerotinia* to pesticides, increasing the difficulty of control, but also leads to excessive accumulation of harmful substances in the soil, reducing soil fertility and causing soil pollution (Sharma et al., 2024; Zuo et al., 2022). Populations of *Sclerotinia* with resistance to benzimidazole fungicides (such as carbendazim) and dimethomorph fungicides (dimethomorphs) are frequently discovered (Duan et al., 2012). Moreover, the excessive use of pesticides in agriculture can cause environmental pollution, and affect ecosystems, soil microbial populations, bacterial diversity, soil enzyme activity, and soil biota, thereby posing a threat to human health (da Silva Júnior et al., 2022; Dewi et al., 2022).

With the increasing emphasis on green agriculture and food safety, various countries have increasingly replaced chemical pesticides with biological control agents (Bailey et al., 2010), especially biocontrol microorganisms, their metabolites, and plant extracts (Ojaghian et al., 2020; Ribeiro et al., 2021). For Sclerotinia disease biocontrol, numerous microorganisms (such as fungi and bacteria) have been proven to induce disease resistance in certain plants (Smolińska and Kowalska, 2018; Han et al., 2023). Zeng et al. (2012) found that *Trichoderma* spp. could effectively protect soybeans from *Sclerotinia* infection and significantly reduced the disease index under field conditions. Ribeiro et al. (2021) found that bacteria isolated from the roots of rapeseed could effectively induce systemic resistance in host plants against *Sclerotinia*.

*Streptomyces* are gram-positive bacteria that can grow in a variety of environments (Alam et al., 2022). Their mycelial morphology is similar to that of fungi. The most remarkable feature of *Streptomyces* is their ability to produce a variety of biologically active secondary metabolites, including antifungal, antiviral, antitumor, and antihypertensive compounds, mainly comprising antibiotics and immunosuppressants (Alam et al., 2022; Parra et al., 2023). Numerous reports have demonstrated that *Streptomyces* exhibits strong biocontrol efficacy against plant pathogenic fungi, including *Phytophthora capsici*, *Fusarium oxysporum and Magnaporthe oryzae.* (Le et al., 2021; Song et al., 2023; Veilumuthu et al., 2024). However, no studies have yet been conducted on the exclusive use of *Streptomyces* for the management of sunflower sclerotinia disease.

*S. ahygroscopicus* var. gongzhulingensis strain 769 is an soil isolated *Streptomyces* strain with broad-spectrum antifungal activity and low toxicity to humans and animals (Du et al., 2020, 2021). In recent years, it has been found that *S. ahygroscopicus* var. gongzhulingensis strain 769 metabolites (S769) showed good control effects on various plant diseases, such as corn large spot disease, sorghum loose smut, rice blast, and watermelon fusarium wilt, and can promote the growth of various crops, including rice, soybeans, and watermelons (Ge et al., 2024).

In the present study, we aimed to investigate the control efficacy of S769 against sunflower sclerotinia rot and its effects on sunflower biomass. First, the inhibitory activity of S769 against *Sclerotinia sclerotiorum* was assessed through *in vitro* antagonism assays. Subsequently, detached leaf and pot experiments were conducted using two application methods: soil mixing (S769-Ms) and root drenching (S769-i), to evaluate their effectiveness in controlling sunflower sclerotinia rot and their impact on defense enzyme activities in sunflower plants and rhizosphere soil. Field trials were then carried out to assess the practical agronomic value of S769. Finally, integrated analyses—including rhizosphere microbiome profiling, root transcriptome sequencing, and quantitative detection of *S. sclerotiorum* in sunflower tissues—were performed specifically for the S769-Ms treatment to elucidate the underlying mechanisms.

## 2 Materials and methods

### 2.1 Plant varieties and strains

The *Streptomyces* strain used in this study was *S. ahygroscopicus* var. gongzhulingensis strain 769, which was preserved and provided by the Microbiology Team of Jilin Academy of Agricultural Sciences. The liquid medium selected was modified Gao’s No. 1 medium: 5 g of peptone, 5 g of sodium chloride, 10 g of glucose, 10 g of soluble starch, and distilled water to 1000 mL.

*Sclerotinia sclerotiorum* was isolated from the lesion margins of a diseased sunflower (*Helianthus annuus*) plant collected in a naturally infected field at Jilin Academy of Agricultural Sciences (124°02’48” E, 43°11’21” N, China, in 2020), purified through single-conidium isolation, pathogenicity-confirmed by fulfilling Koch’s postulates, and molecularly identified via ITS and RPB2 gene sequencing with corresponding sequences deposited in NCBI under accession numbers PX103168 and PX111094. The fungal isolate was initially cultured on potato dextrose agar (PDA) (Haibo Biotechnology Co., Ltd., Qingdao, China) at 26 °C for 14–21 days, with relative humidity maintained at 85% under dark conditions.

The sunflower variety used was “Jikuiza 20”, kindly provided by the Peanut Research Institute of Jilin Academy of Agricultural Sciences. This variety is susceptible to *S. sclerotiorum* infection.

The *Sclerotinia*-infested soil was collected from the mature *Sclerotinia*-infested field of the Peanut Research Institute of Jilin Academy of Agricultural Sciences. The soil and diseased plant residues in this field contain overwintering sclerotia and have been stably infected for three consecutive years.

### 2.2 Preparation of S769

Corn grits purchased from the market were used as the culture medium. The medium sterilized at 121 °C for 60 min, *S. ahygroscopicus* var. gongzhulingensis was inoculated (300 g corn grits were inoculated 10 mL of 1×10^6^ CFU/mL Gao’s No. 1 *S. ahygroscopicus* var. gongzhulingensis fungal suspension) and the culture was carried out at 28 °C for 7 d. The activity was detected after the gray powder was formed on the surface of the corn ballast culture medium, and the number of target fungus reached more than 1×10^6^ CFU/mL and was considered sufficiently cultured. The corn grit cultures were spread on cheesecloth-lined trays in a light-deprived chamber for shade drying. Upon achieving ambient humidity below 30%, the desiccated cultures were pulverized and sieved through a 100-mesh screen for storage (Wu and Pang, 1979). The S769 was extracted from the stored dry powder through mixing with pure water at a mass-to-volume ratio of 1:2 and incubation at 4 °C for 24 h. The mixture was centrifuged at 2000 rpm 5min and filtered to obtain the supernatant, which was termed S769 (Wu and Pang, 1979). The inhibitory activity of S769 against pathogens was detected using northern corn leaf blight (*Setosphaeria turcica*) as the indicator organism. An inhibition zone diameter ≥3.0 cm indicated that the culture’s biological activity met application requirements.

### 2.3 *In vitro* antagonism experiment

The *in vitro* antagonistic activity of S769 against *S. sclerotiorum* was tested using the three–point confrontation culture method (Zhang et al., 2022). *S. sclerotiorum* cultures that had been grown for 15 d were used. A 5 mm diameter mycelial disc was prepared from the edge of the colony using a punch and inoculated at the center of a potato dextrose agar medium plate. Sterile Oxford cups (Ningbo Ja-Hely Technology Co., Ltd) were positioned bilaterally on the mycelial disc at fixed 1.5 cm intervals, with symmetrical placement relative to the colony center. Each treatment had 10 biological replicates, and the plates were then incubated at 26 °C under a 12-h photoperiod. Then, 150 μL of S769 (extracted as described in section 2.2) was added to each Oxford cup. Six treatments were set up: (1) A control treatment with only *S. sclerotiorum* inoculated (Control); (2) S769 (G); (3) a 50-fold dilution of S769 (G–50X); (4) a 100-fold dilution of S769 (G-100X); (5) a 200-fold dilution of S769 (G-200X); (6) a 400–fold dilution of S769 (G-400X). Treatments 2–6 were also inoculated with *S. sclerotiorum.* Each treatment was repeated three times, with five plates per repetition. The plates were incubated at a constant temperature of 26 °C for 5 d, The inhibitory activity was evaluated based on the inhibition of fungal pathogens growth, and the diameter was measured at 7 dpi, the mycelial radial growth of *S. sclerotiorum* on a control plate and in the direction of S769 was measured.

### 2.4 Detached leaf experiment

Three treatments were set up in this experiment: (1) Untreated plants as the blank control (Control); (2) plants treated with dry powder of *Streptomyces* culture mixed with soil at sowing (S769-Ms); and (3) plants treated with a 100-fold dilution of S769 by root irrigation (S769-i). Each treatment was repeated five times, with eight leaves per repetition.

The specific experimental method referred to Sui et al (Sui et al., 2025). Healthy sunflower seedlings with consistent growth conditions were selected from each of the three treatment groups. The middle leaves of the seedlings were gently washed and dried. The leaves were then placed flat on filter paper. Mycelial blocks of uniform size (5 mm diameter) were prepared from the edge of a 15-day-old *S. sclerotiorum* culture plate, 2 cm away from the edge. The mycelial part of the blocks was closely attached to the leaves (avoiding contact with the veins), and the petioles were wrapped with moist sterile cotton to maintain humidity. The treated plates were placed in an artificial climate chamber at 26 °C and subjected to a 16 h light and 8 h dark cycle. After the plants showed symptoms of disease, the diameter of the lesions was measured with Vernier calipers, and the incidence rate was recorded.

### 2.5 Evaluation of the control effect of S769 on sunflower rot

In circular planting pots with a diameter of 12 cm and a height of 18 cm, 300 g of *Sclerotinia* rot-infected soil was filled. Two seeds of Jikuiza 20, which had been pre–germinated, were sown in each pot, and a consistent amount of water was applied. After the seeds germinated, one seedling was reserved in each pot. Three treatment groups were then set up: (1) No treatment was applied as the control group (Control); (2) 8 g of dry powder of *Streptomyces* culture was mixed with the infected soil before sowing as the soil–mixing treatment group (S769-Ms); (3) the roots of seedlings with four true leaves were drenched with 40 mL of a 100-fold dilution of S769 per seedling for three times with 24 h intervals as the root-drenching treatment group (S769-i). Each treatment consisted of 10 potted sunflower seedlings with three biological replicates.

The disease index was classified according to the method of Zhang et al (Zhang and Xue, 2010): Grade 0: no symptoms; grade 1: a few necrotic or diseased spots on the leaves, with a total disease area< 5%; grade 2: multiple lesions on the leaves, with a total disease area of 5–10%; grade 3: numerous lesions on the leaves, with a total disease area of 11-30%, and stem rot; grade 4: large lesions on the leaves, with a total disease area of 31-50%, and stem rot; and grade 5: large lesions on the leaves, with a total disease area of over 51%, and stem rot or plant death. The disease index was calculated using the following formula:


$$\text{Disease index }\left(\%\right)=\sum \frac{\text{(The number of disease level }\times \text{ the corresponding disease level);}}{\text{(Total number of investigated plants }\times \text{ highest representative level)}}\times 100$$

(Song et al., 2004).

The effects of different treatments on the growth characteristics of sunflowers were investigated during their growth process.

### 2.6 Effects of S769 on the activity of defense enzymes in sunflower plants and soil

Samples were collected on the final day of disease severity rating. Five sunflower plants from each treatment group (as described in Section 2.5) were randomly selected. Two leaf samples were collected from each plant and wrapped in tin foil for rapid freezing in liquid nitrogen. At the same time, rhizosphere soil samples from the same plants in each treatment group were collected, air-dried, ground, and sieved (60–80 mesh). All samples were stored at -20 °C for subsequent determination of plant enzyme activities and soil enzyme activities.

The activity of superoxide dismutase (SOD) was determined using the xanthine and xanthine oxidase (XO) method (Ukeda et al., 1999), the activity of polyphenol oxidase (PPO) was determined using the o-diphenol method (Tang and Newton, 2004), and the activity of catalase (CAT) was determined using the ultraviolet spectrophotometric method (Zhang et al., 2018). All experiments were repeated three times.

Urease activity was determined using the phenol-sodium hypochlorite colorimetric method (Tavares et al., 2021), invertase activity was determined using the 3,5-dinitrosalicylic acid colorimetric method (Visvanathan et al., 2021), and acid phosphatase (ACP) activity was determined using the sodium phenyl phosphate colorimetric method (Olougou et al., 2024). All experiments were repeated three times.

### 2.7 Field experiment

The treatment design was the same as that described in Section 2.5. The experiment was conducted at the Gongzhuling Base of the Jilin Academy of Agricultural Sciences. Three biological replicates were conducted for each treatment, with each replicate plot (72 m²) containing 450 sunflower seedlings. At the flowering stage (35 days after sowing) and the maturity stage (85 d after sowing), two field investigations were conducted through random sampling to ensure consistency in the plants before and after. At the maturity stage, several sunflower plants were randomly selected as samples to investigate the 100-grain weight, 1,000-grain weight, and the shriveled grain rate of the seeds. The sunflower heads were picked and the plump seeds and shriveled grains were separated and counted respectively. Plump seeds refer to those that are normally developed and fully filled; shriveled grains are those that are poorly developed, shriveled, and not plump.

The formula to calculate the percentage of shriveled grains was:


$$\text{Percentage of shriveled grains}\left(\%\right)=(\frac{\text{Number of shriveled grains}}{\text{Total number of grains}})\times 100$$

(Gu, 2013).

After randomly mixing the above seeds, they were weighed to obtain the results of the 100–grain weight, and the 1,000-grain weight.

The disease index was observed using the grading system mentioned in section 2.2.4.

### 2.8 Detection of the *S. sclerotiorum* content in sunflowers using quantitative PCR

On the 40th day after sowing in the pot experiment, samples of leaves and lateral roots were collected from the S769-Ms treatment group and the Control treatment group. The sample disinfection method was based on that reported by Sui et al (Sui et al., 2025), and the excess surface water was removed using filter paper. The samples were then wrapped in tin foil, rapidly frozen in liquid nitrogen, and stored at -80 °C. Leaf and root tissues from three biological replicates were pooled and homogenized separately. and total RNA was extracted from the plant roots using the Cetyltrimethylammonium bromide method (Ueno et al., 2024). The presence of the pathogen in the seedlings was quantified using quantitative PCR using specific primers, according to the description of Sui et al (Sui et al., 2025). For this experiment, Actin was selected as the reference gene with three technical replicates performed, and the qPCR methodology employed relative quantification (ΔΔCt method) to determine copy number.

### 2.9 Effects of S769 on the soil microbiome and the plant transcriptome

The sunflower roots used in this experiment were the same as those described in Section 2.8. Total RNA was extracted from the sunflower roots, and its concentration and quality were detected using a micro-nucleic acid protein detector (Nanodrop, Thermo Fisher Scientific, Waltham, MA, USA). After confirmation, the samples were sent to Beijing Novogene Bioinformatics Technology Co., Ltd. for high-throughput amplicon sequencing of the bacterial community in rhizosphere soil performed on the Illumina NovaSeq 6000 platform (Illumina Inc. San Diego, CA, USA) (Ge et al., 2024), and paired-end libraries were sequenced on the Illumina HiSeq X Ten platform (Ge et al., 2024) after PCR amplification to enrich the library fragments.

The raw sequencing data were analyzed using USEARCH in QIIME version 1.9.0 (Caporaso et al., 2010). Zero-radius operational taxonomic units (ZOTUs) were identified using UNOISE3 with 100% similarity, and chimeras were removed (Edgar, 2010). The representative sequences of bacterial ZOTUs were annotated using BLAST, referencing the SILVA (version_132) and UNITE (version_7.2) databases. The classification of bacteria and their components at various taxonomic levels was performed using the script *summarize_taxa.py*. The α–diversity (including ZOTU richness and the Shannon index) and β-diversity (based on Weighted-UniFrac distance) of the microorganisms were calculated using the scripts *alpha_diversity.py* and *beta_diversity.py*, respectively. Integrity of root system RNA was evaluated using the RNA Nano 6000 Assay Kit in conjunction with the Agilent 2100 Bioanalyzer system (Agilent Technologies, Santa Clara, CA, USA). Total RNA served as the input material for RNA sample preparation. Specifically, mRNA was purified from total RNA using poly-T oligo-attached magnetic beads (Illumina, inc. San Diego, CA, USA). Fragmentation was conducted using divalent cations under elevated temperature conditions in the First Strand Synthesis Reaction Buffer (5×). First-strand cDNA was synthesized using random hexamer primers and M-MuLV Reverse Transcriptase (RNase H-) (New England Biolabs, Inc., USA).

Subsequently, The second-strand cDNA synthesis was performed using DNA Polymerase I (New England Biolabs, Inc., USA) and RNase H (New England Biolabs), followed by blunt-end conversion of overhangs via exonuclease/polymerase activities. After 3’ end adenylation, hairpin loop adaptors were ligated for hybridization. Library fragments were size-selected (370–420 bp) using AMPure XP purification, followed by PCR amplification with Phusion High-Fidelity DNA polymerase (New England Biolabs) and custom primers (Integrated DNA Technologies).

Finally, PCR products underwent AMPure XP purification followed by library quality assessment via Agilent Bioanalyzer 2100. Index-coded samples were clustered on a cBot system using TruSeq PE Cluster Kit v3-cBot-HS (Illumina), followed by 150 bp paired-end sequencing on Illumina Novaseq. Raw data (6 GB/sample) were processed to remove adapters and low-quality reads, with Unique Molecular Identifiers (UMIs) trimmed and clustered to eliminate redundancy. Clean reads were mapped to the sunflower reference genome (GCF_002127325.2) using HISAT2 v2.0.5 (https://daehwankimlab.github.io/hisat2/), with an average mapping rate of 85%, and gene expression was quantified via HTSeq (0.9.1) with FPKM normalization. Sequence reads were aligned to the reference genome using Bowtie2 (v2.4.5), and transcript abundances were quantified via Express (v1.5.1). Differential expression analysis was conducted with DESeq under stringent thresholds (|log_2_FC|>1, adjusted *P*<0.05). All *P*-values were adjusted for multiple testing via the Benjamini-Hochberg FDR procedure, and genes with FDR< 0.05 were considered significant. Significantly differentially expressed genes (DEGs) were subsequently subjected to functional annotation through Gene Ontology (GO) enrichment and KEGG (Kyoto Encyclopedia of Genes and Genomes) pathway mapping using the clusterProfiler R package (v4.0), with significance defined by corrected *P*<0.05.

### 2.10 Data analysis

One-way analysis of variance followed by the Duncan test was conducted to compare the differences among three treatments using SPSS 25.0 (IBM Corp., Armonk, NY, USA). GraphPad Prism 8.0.2 (GraphPad Inc, La Jolla, CA, USA) was used for data visualization. Principle coordinate analysis of bacterial and fungal communities based on the Weighted–Unifrac distance was applied to visualize the differences in microbial communities, and the significance among different treatments was tested using permutational analysis of variance in the veganpackage in R. The difference in microbial composition at the phylum or class level between each two treatments was analyzed using the Wilcoxon test at *P<* 0.05. Linear discriminant analysis Effect Size (LEfSe) analysis at the genus level was conducted to identify the microbial taxa enriched by continuous cropping, and the genera with logarithmic linear discriminant analysis score >3.0 and Wilcoxon test at *P*< 0.05 were considered as the biomarker taxa.

## 3 Results

### 3.1 Inhibitory effects of different concentrations of S769 on *S. sclerotiorum*

**Figure 1:**
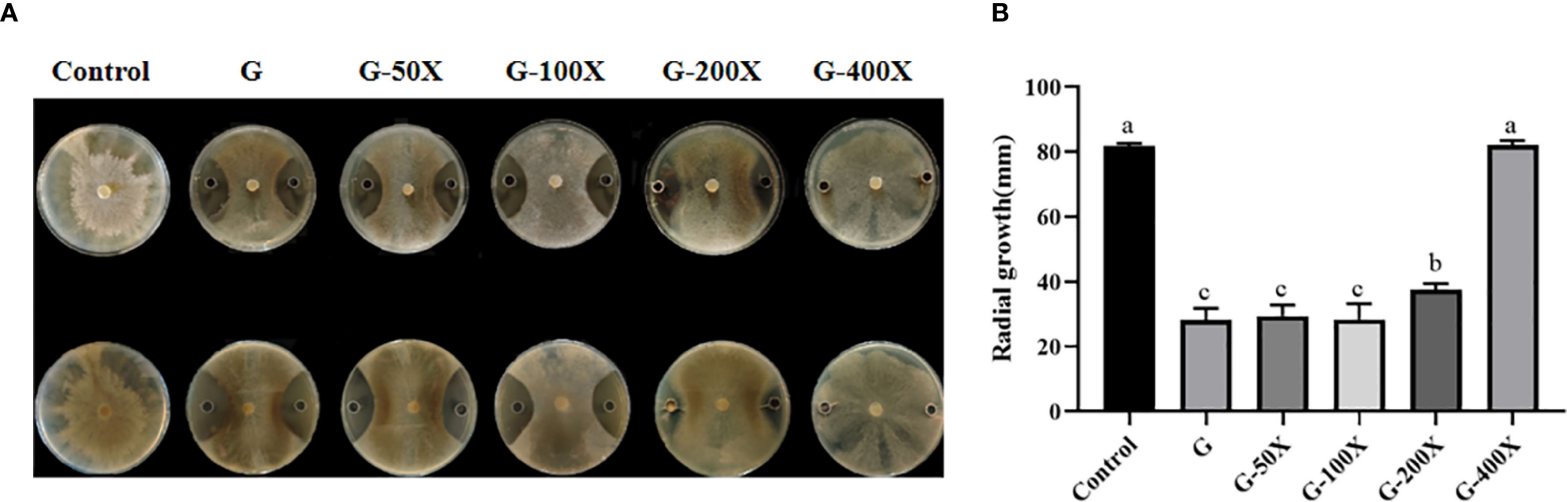
The antibacterial effects of different concentrations of S769. **(A)** Appearance of the plates after 10 days of confrontation culture. **(B)** The radial diameters of *S. sclerotiorum* colonies measured on the 10^th^ day of plate confrontation culture in the control group and the G, G-50X, G-100X, G-200X, and G-400X treatment groups. Different letters indicate significant difference in different treatments and compartments at *p*< 0.05 by one-way ANOVA. The data are presented as mean values ± standard deviation (SD).

After 10 d of confrontation culture, the G-400X treatment group showed no inhibitory effect, while the inhibition rates of the G, G-50X, G-100X, and G-200X treatment groups against *S. sclerotiorum* were 65.51%, 64.15%, 65.79%, and 54.07%, respectively (**Figure 1A**). Statistical analysis indicated that there was no significant difference among the G, G–50X, and G-100X treatment groups (*P* > 0.05, **Figure 1B**); however, the inhibitory effect of the G-200X treatment was significantly weaker than that of the former three. Therefore, to achieve efficient utilization of S769 and reduce its potential phytotoxic effects, the G–100X concentration was selected for subsequent experiments.

### 3.2 Inhibitory effect of S769 on *S. sclerotiorum* infection *in vitro*

**Figure 2:**
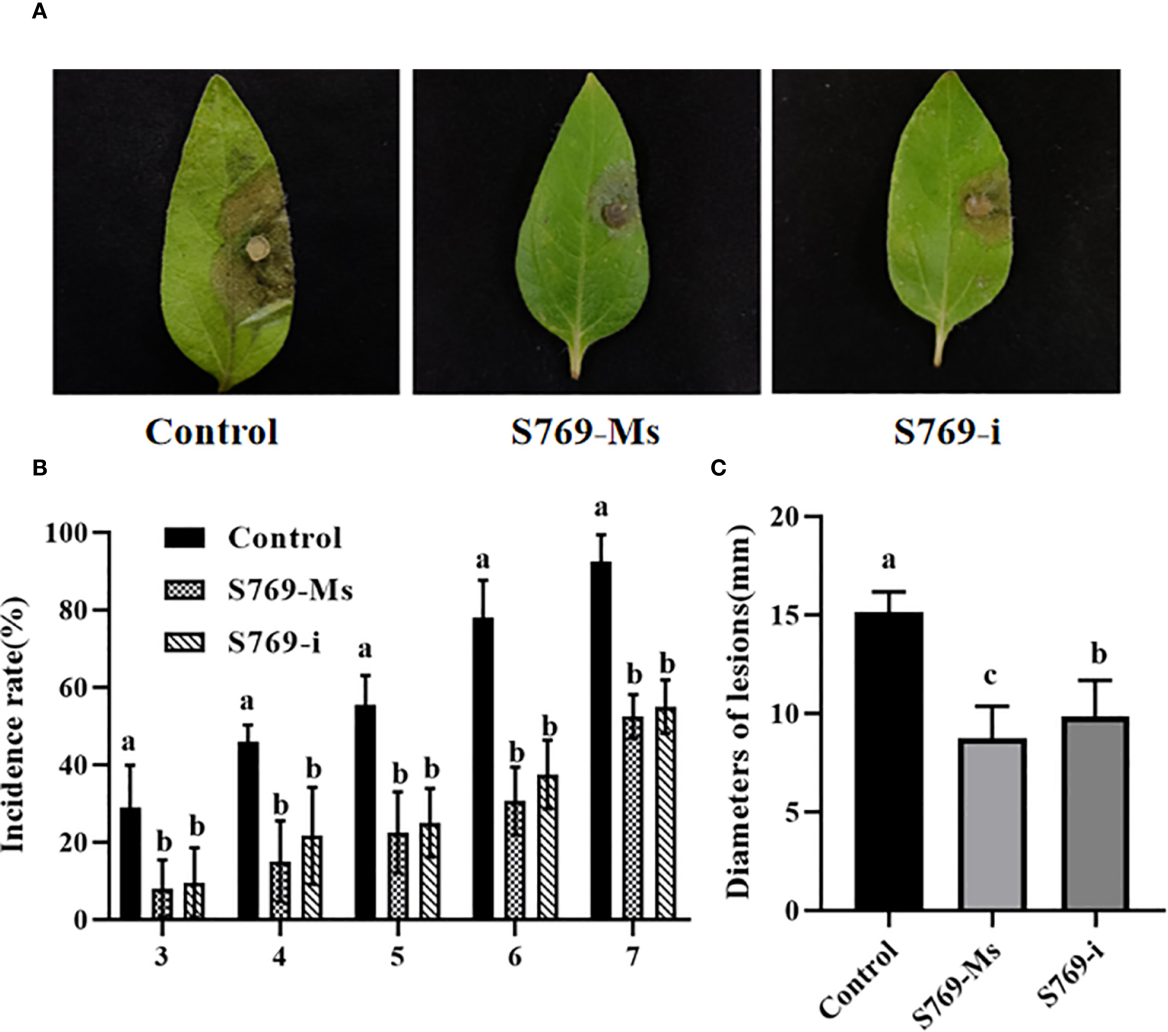
Lesions on leaves the 5^th^ day post-inoculation with *S. sclerotiorum*. **(A)** The disease morphology. **(B)** Disease incidence. **(C)** Lesion diameter of sunflower leaves in the different treatment groups at various time points. Different letters indicate significant difference in different treatments and compartments at *p*< 0.05 by one-way ANOVA. The data are presented as mean values ± standard deviation (SD).

In the *in vitro* sunflower leaf experiment, symptoms of disease began to appear in all treatment groups on the third day after inoculation, and the incidence rate showed an increasing trend with time. Among the groups, the incidence rate of the control group was always significantly higher than that of the experimental groups treated with S769. 5 d after inoculation of *S. sclerotiorum* on sunflower leaves, large areas of lesions appeared on the leaves of the control group, accompanied by significant leaf yellowing and rotting. In contrast, only a few lesions were observed in the S769-Ms and S769-i treatment groups (**Figure 2A**). On the seventh day after inoculation of *S. sclerotiorum*, the incidence rate of the control group was 92.53%, while the incidence rates of the S769-Ms and S769–i treatment groups were 52.53% (*P<* 0.001, F = 102.400) and 55.16% (*P<* 0.001, F = 75.000), respectively (**Figure 2B**). The results of lesion diameter measurement also indicated that the S769 treatment groups could effectively prevent the spread of lesions (**Figure 2C**). The lesion diameter of the control group was 15.12 ± 1.05 mm, while the lesion diameter of the S769-Ms treatment group was significantly reduced to 8.63 ± 1.34 mm (*P<* 0.001, F = 585.362), and that of the S769-i treatment group was 9.73 ± 1.61 mm (*P<* 0.001, F = 314.317).

### 3.3 Effects of S769 induced resistance to sunflower sclerotinia rot in pot experiment

**Figure 3:**
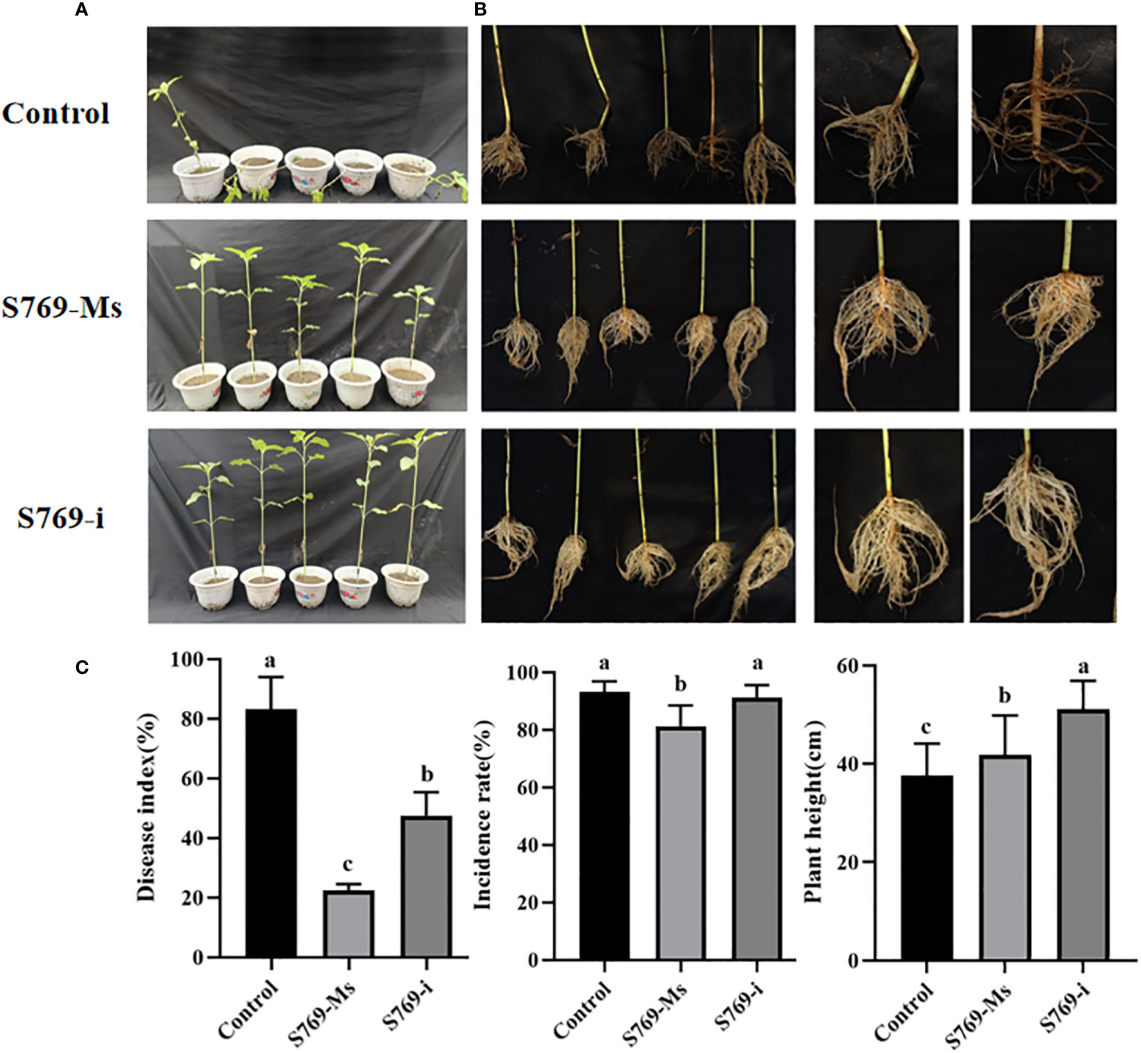
Disease incidence in sunflower plants potted in *S. sclerotiorum*-infested soil in the different treatment groups. **(A)** Disease incidence on the 40^th^ day. **(B)** Condition of the root system of diseased plants. **(C)** Disease incidence, disease index, and plant height of sunflower plants potted in *S. sclerotiorum*-infested soil in the different treatment groups on the 40^th^ day. Different letters indicate significant difference in different treatments and compartments at *p*< 0.05 by one-way ANOVA. The data are presented as mean values ± standard deviation (SD).

The disease indexes of sunflower seedlings in the pot experiment at 40 d after sowing in diseased soil are shown in (**Figure 3A**). In the control group, the sunflower leaves showed extensive wilting and grayish lesions, accompanied by lodging. In contrast, the sunflower seedlings treated with S769-Ms and S769-i showed only a few lesions and no lodging (**Figure 3B**). The disease incidence was 96.88% in the control group, 81.25% in the S769-Ms group (*P<* 0.05, F = 10.714, **Figure 3C**), and 93.75% in the S769-i group (*P* > 0.05, F = 0.429). The disease index was 83.13% in the control group, 22.51% in the S769-Ms group (*P<* 0.001, F = 120.115), and 47.53% in the S769-i group (*P<* 0.05, F = 28.089). After the sunflower seedlings were removed from the diseased soil and thoroughly washed, it was observed that the stems of the plants in the control group had large lesions, accompanied by hollowing, breaking, and rotting of the roots and stems, while the seedlings in the two S769 treatment groups showed good growth. The seedling height was 37.55 ± 6.54 cm in the control group, 41.77 ± 8.06 cm in the S769-Ms group (*P<* 0.05, F = 5.271), and 51.14 ± 5.75 cm in the S769-i group (*P<* 0.001, F = 77.944). These results indicated that the application of S769 could significantly reduce the incidence of sunflower sclerotinia stem rot and delay the infection process to a certain extent. Additionally, S769 treatment promoted the growth of sunflower seedlings.

### 3.4 Effects of S769 on defense enzyme activities in sunflower seedlings and soil

**Figure 4:**
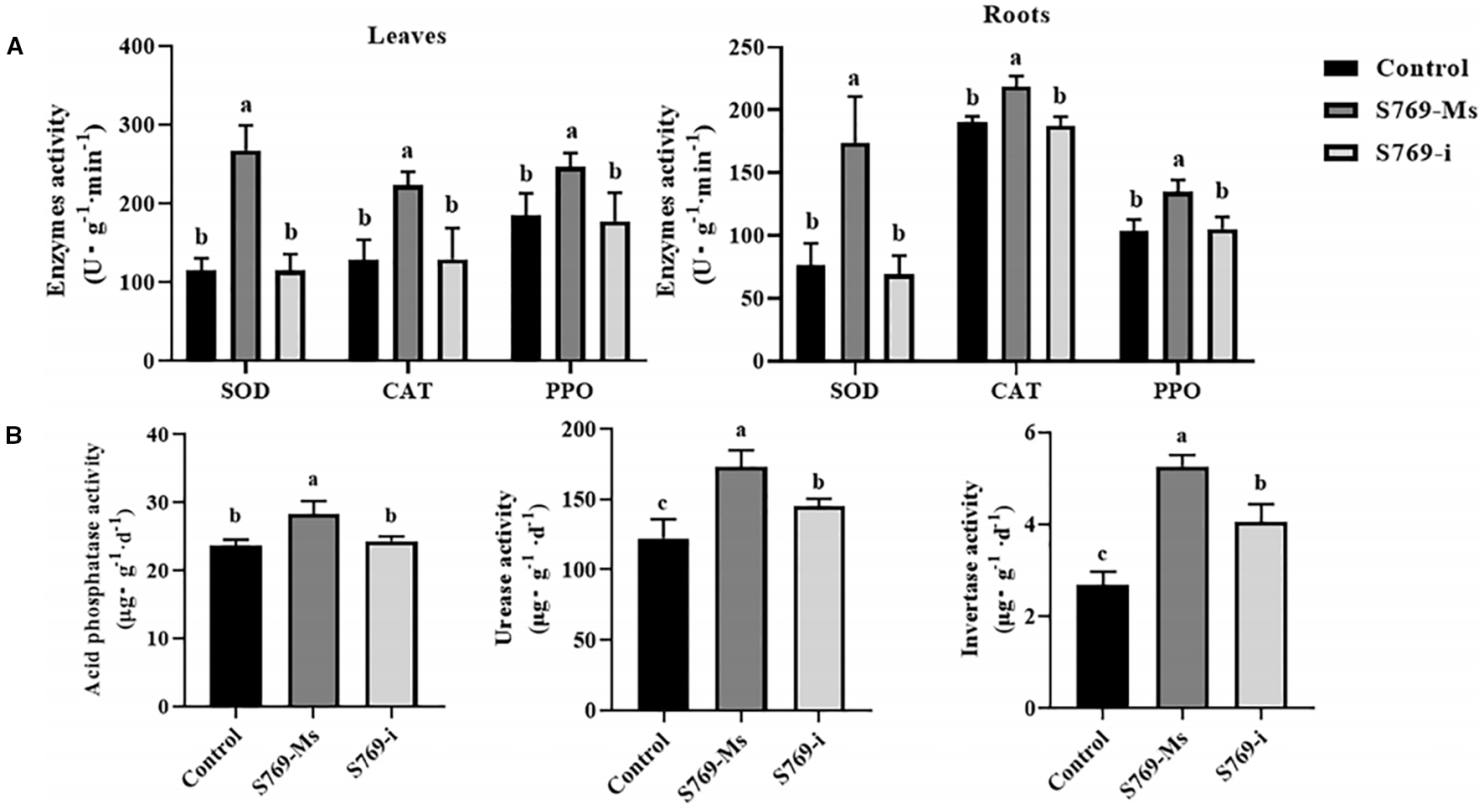
Effects of different treatments on enzyme activities after 40 days in *S. sclerotiorum*-infested soil. Different letters indicate significant difference in different treatments and compartments at *p*< 0.05 by one-way ANOVA. The data are presented as mean values ± standard deviation (SD). **(A)** SOD, CAT, and PPO activities in the leaves and roots of sunflower plants. **(B)** The activities of sucrase, acid phosphatase, and urease in the rhizosphere soil of sunflower under different treatment conditions.

Compared with those in the control group and the S769-i group, the activities of SOD, CAT, and PPO in the leaves and roots of sunflower plants in the S769-Ms group were significantly increased (*P<* 0.05, **Figure 4**). Additionally, S769-Ms treatment significantly affected the enzyme activities in the rhizosphere soil of sunflower plants (**Figure 4**). The activities of soil acid phosphatase, urease, and sucrase in the S769-Ms group were 19.70%, 29.49%, and 49.05% higher than those in the control group, respectively, and were 14.04%, 16.15%, and 22.81% higher than those in the S769-i group, respectively. Statistical analysis indicated that there were significant differences between the S769-Ms group and the other two groups (*P<* 0.05). The results demonstrated that S769-Ms not only enhanced the activities of defense enzymes in sunflower seedlings, but also effectively improved the rhizosphere soil enzyme activities.

### 3.5 Effects of S769 on sclerotinia rot and seeds of sunflowers in the field experiment

**Figure 5:**
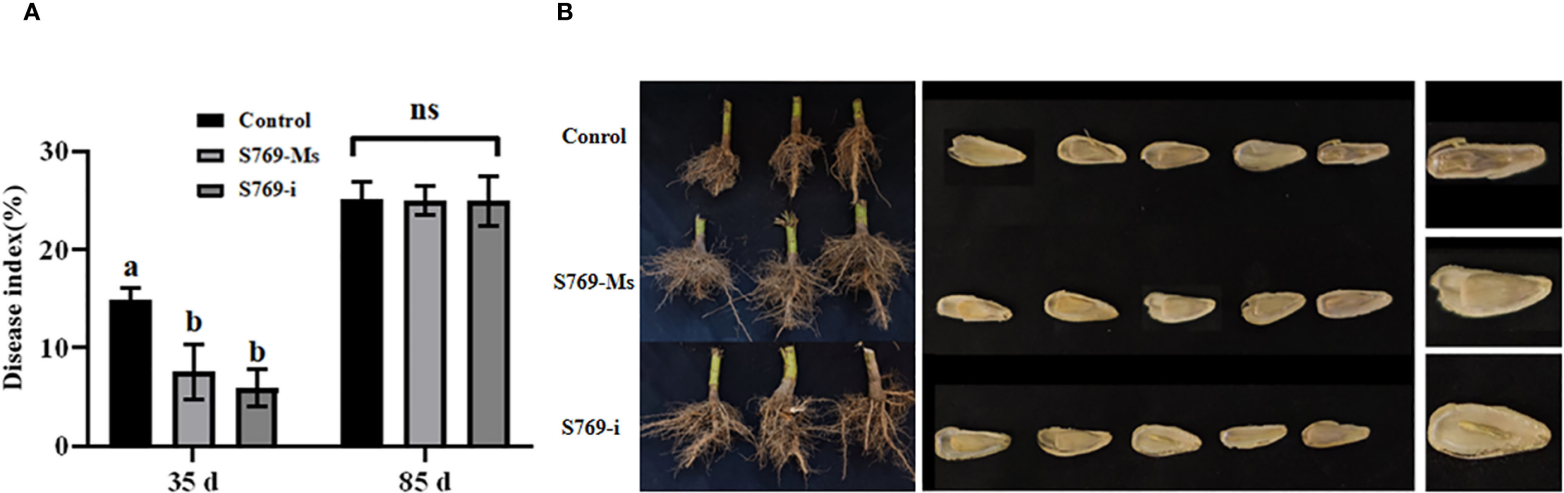
Disease index of sunflower plants infected with *S. sclerotiorum* in the different treatment groups. **(A)** Disease index on the 35^th^ and 85^th^ day. Different letters indicate significant difference in different treatments and compartments at *p*< 0.05 by one-way ANOVA. The data are presented as mean values ± standard deviation (SD). **(B)** Root condition and seeds status of the different treatment groups.

At the 35th day, the disease index of the control treatment group reached 14.69%, while the disease index of the S769-Ms treatment group was significantly reduced to 7.36% (*P<* 0.05, F = 26.936), and the disease index of the S769-i treatment group was further reduced to 5.92% (*P<* 0.001, F = 80.639). However, by the 85th day, the disease indexes of each treatment group tended to be consistent, showing no significant difference (*P* > 0.05) (**Figure 5A**). The results indicated that, compared with the control group, the sunflower plants in the S769 treatment group exhibited significant differences in root development characteristics. Regarding seed plumpness, the seeds in the control group were noticeably underdeveloped, and the proportion of shriveled fruits was higher than that in the treatment groups (**Figure 5B**). The fresh weight of the roots in the control treatment group was 428.11 ± 42.71, and the fresh weight of the roots in the S769-Ms treatment group was 494.13 ± 61.41 g (*P<* 0.001, F = 35.059). At the 85th day after sowing, the results of sunflower plant height, root fresh weight, yield and shriveled seed rate (**Table 1**) showed that compared with the control treatment group, the fresh weight of the roots of sunflowers treated with S769-Ms increased by approximately 13.36%, and the shriveled seed rate decreased by approximately 31.49%. The results indicated that the root biomass of sunflower plants treated with S769-Ms was significantly higher than that of the control treatment group. During the field control experiment, S769 could effectively control *S. sclerotinia* infection in the early stage.

**Table 1:** Effect of S769 on the growth of sunflowers in the field.

Treatment	Plant height (cm)	Root fresh weight (g)	Hundred-grain weight (g)	Thousand-grain weight (g)	Shriveled-grain rate (%)
Control	190.11 ± 10.12b (**210.73**)	428.11 ± 42.71b (**516.96**)	16.9 ± 1.37a (**27.73**)	169.2 ± 10.17a (**184.66**)	28.9 ± 5.51b (**34.14**)
S769-Ms	187.63 ± 11.75b (**207.35**)	494.13 ± 61.41a (**584.17**)	18.2 ± 1.23a (**31.84**)	173.4 ± 11.42a (**191.19**)	19.8 ± 8.16a (**29.67**)
S769- i	195.82 ± 12.16a (**212.26**)	495.96 ± 51.15a (**594.49**)	17.6 ± 0.35a (**29.21**)	177.6 ± 9.05a (**188.87**)	19.0 ± 7.99a (**29.32**)

The data represent the means ± SD (standard deviation). The data in bold in the table represents the maximum value for that item.

Different letters in the same column indicate significant differences (*P<* 0.05) in each treatment according to Duncan’s multiple range test.

### 3.6 Effects of S769 on the rhizosphere microbiome of sunflower seedlings

**Figure 6:**
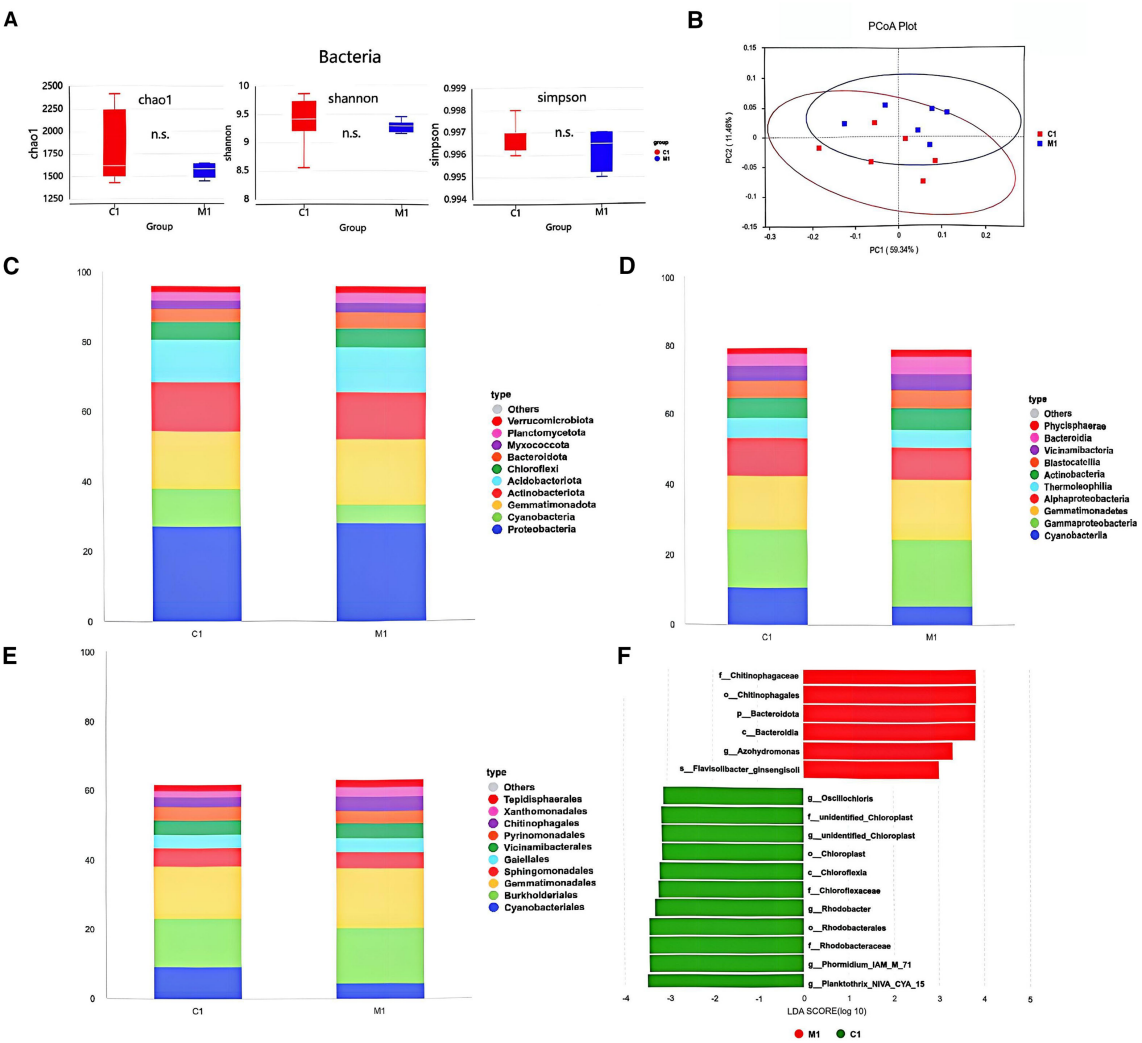
**(A)** Effects of soil treatment with S769 (M1) on alpha diversity (*P* > 0.05). **(B)** Principal coordinate analysis plots were used to visualize and compare variations between treatments based on the Weighted UniFrac distance of bacterial communities. The differences in soil bacterial community composition among different treatments in the field experiment. Histograms depict the relative abundance of bacterial communities at the phylum level **(C)**, class level **(D)**, and order level **(E)** (relative abundance > 1%). **(F)** Microbial biomarkers in the two treatments as revealed by LEfSe analysis.

The rhizosphere soil samples treated with S769-Ms and the control samples showed high similarity in terms of species composition structure and community structure. Alpha diversity analysis, including Chao1, Shannon, and Simpson indices, indicated that the application of S769 had no significant effect on the diversity and richness of the bacterial community in the root soil of the plants (**Figure 6A**). Principal coordinate analysis of the bacterial community showed that treatment with S769 had no significant impact on the structure of the microbial community (**Figure 6B**). At the phylum level (**Figure 6C**), treatment with S769 increased the relative abundance of Proteobacteria (*P* = 0.017) and Actinobacteria (*P* = 0.032) but decreased that of Cyanobacteria (*P<* 0.05). At the class level (**Figure 6D**), γ–Proteobacteria and Actinobacteria became the dominant classes; while at the order level, Burkholderiales and Actinomycetales were dominant (**Figure 6E**). Through LEfSe analysis (**Figure 6F**), it was further determined that the Chitinophagaceae (*P* = 0.006), Bacteroides (*P* = 0.029), Nitriliruptoraceae (*P* = 0.045), *Flavobacterium* (*P* = 0.038), and *Sphingomonas* (*P* = 0.043) were the main biomarkers under the S769-Ms treatment (*P*< 0.05).

### 3.7 Transcriptional differences in sunflower plants after treatment with S769

**Figure 7:**
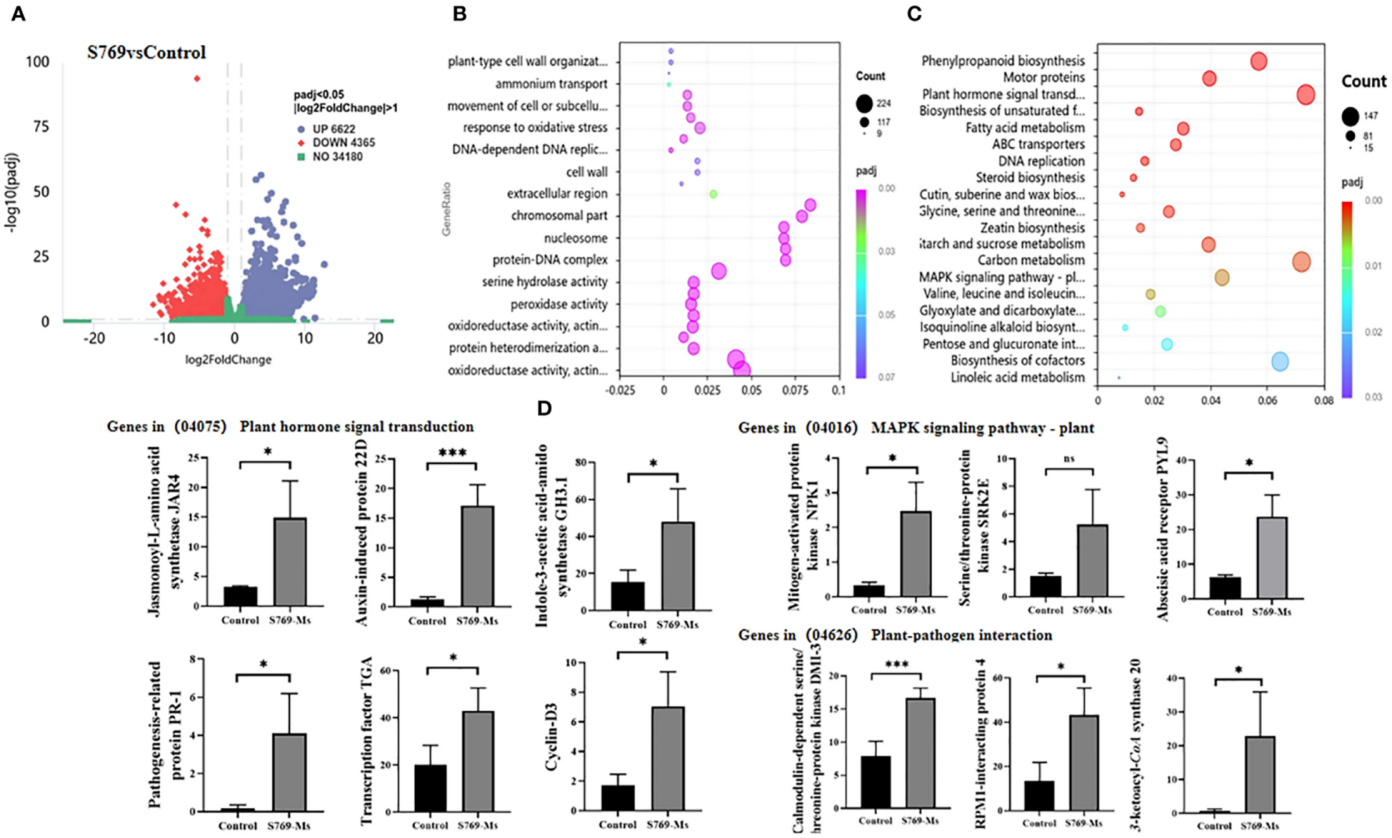
Root transcriptomic variations among different treatment groups. **(A)** Volcano plots visualizing the differentially expressed genes (DEGs) between the S769 treatment and the control groups (|log2FoldChange| > 1, Student’s t-test *P<* 0.05). Each dot represents a gene, with the x-axis indicating the fold change in expression and the y-axis representing the statistical significance (–log10(p–value)). **(B)** Bubble chart showing the expression of DEGs belonging to GO enrichment categories based on Student’s t-test. The x-axis of the bubble chart represents significant differences with *P<* 0.05. **(C)** The biomarkers within the Kyoto Encyclopedia of Genes and Genomes (KEGG) pathways were identified by mapping DEGs to the KEGG database using hypergeometric distribution methods at *P<* 0.05. **(D)** Gene expression levels were standardized using the Fragments Per Kilobase Million method throughout the analysis. The asterisks represent level of significant (* *p*< 0.05, ** *p*< 0.01) between two treatments based on Student’s t-test (n = 3). The data are presented as mean values ± standard deviation (SD).

Through transcriptome sequencing analysis, 43.68 Gb of effective data were obtained. A volcano plot of differential expression between the control group and the S769-Ms treatment group (**Figure 7A**) identified 10,987 DEGs (|log2FoldChange| > 1, *P*< 0.05). Among them, 6,622 DEGs were upregulated and 4,365 DEGs were downregulated after S769-Ms treatment.

Gene ontology (GO) enrichment analysis of the control treatment group and the S769–Ms treatment group (**Figure 7B**) indicated that after S769-Ms treatment, there were significant differences in gene expression in the sunflower root system. The GO functional classification was assessed in three categories: biological processes, cellular components, and molecular functions. In the biological processes category, the DEGs were involved the synthesis of plant cell wall organization. In cellular components, the DEGs were related to protein-nucleic acid complexes. In molecular function, the DEGs were associated with iron ion binding and antioxidant activity processes. GO enrichment analysis showed that S769-Ms treatment had a wide range of regulatory effects on gene expression in sunflower roots, involving key biological processes such as dynamic changes of cytoskeleton (such as microtubule-related processes), DNA replication and cell growth, cell wall synthesis and tissue, and oxidoreductase activity.

In the KEGG pathways (**Figure 7C**), S769-Ms treatment upregulated genes associated with plant hormone signal transduction and mitogen activated protein kinase (MAPK) signaling pathways. Among the downregulated genes, some disease resistance genes in the pathways of interaction with plant pathogens, cysteine and methionine metabolism, and alpha-linolenic acid metabolism were upregulated. In the plant hormone signal transduction pathway, the expression of multiple genes significantly increased under S769-Ms treatment, and some genes in the MAPK signaling pathway and plant-pathogen interaction pathway were significantly upregulated under S769-Ms treatment (**Figure 7D**).

### 3.8 Content of *S. sclerotiorum* in sunflower seedlings

**Figure 8:**
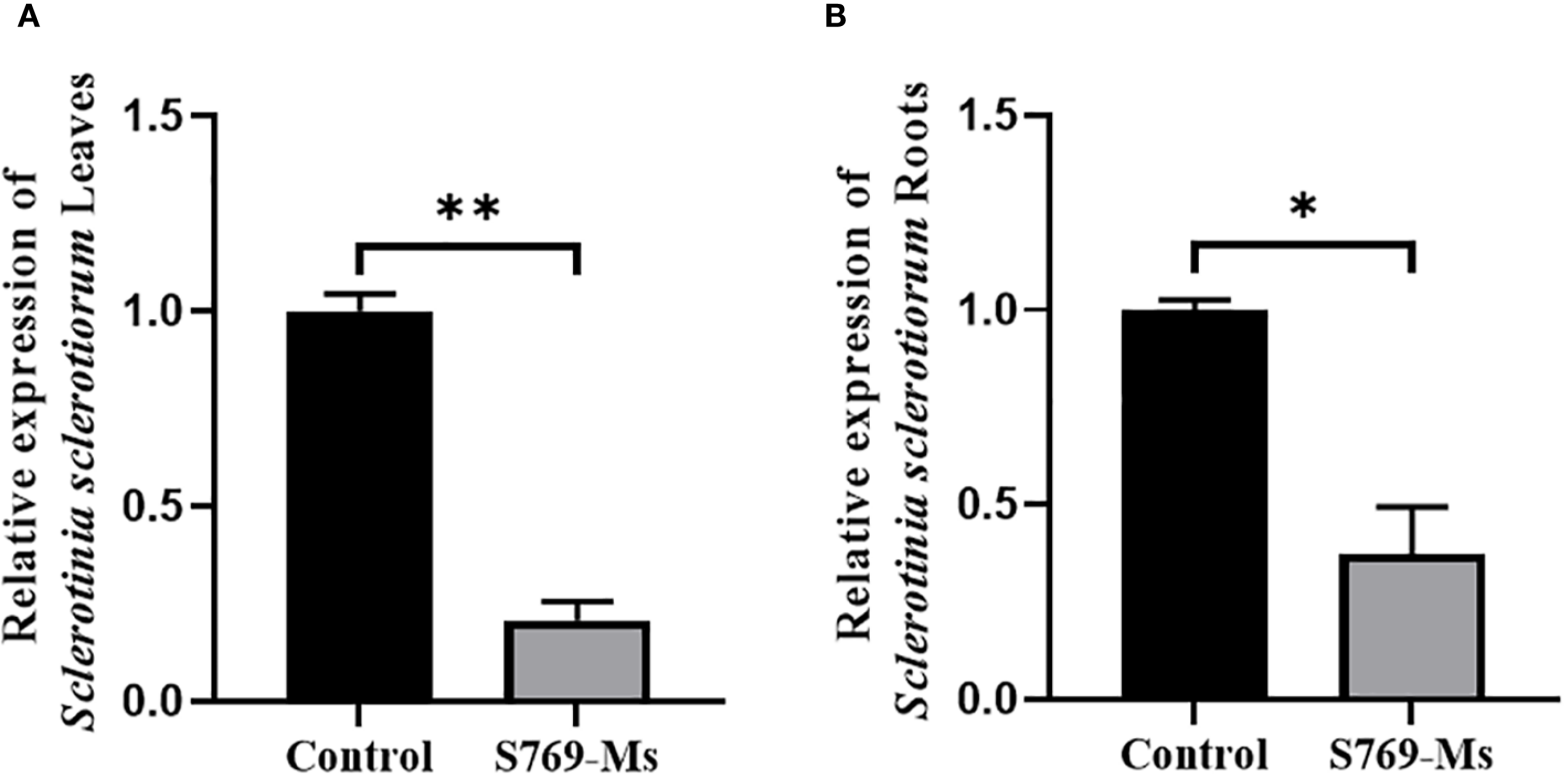
The quantification of *S. sclerotiorum* in the **(A)** leaves and **(B)** roots after application of S769 to the soil. The asterisks represent level of significant (* *p*< 0.05, ** *p<* 0.01) between two treatments based on Student’s *t*-test (*n* = 3). The data are presented as mean values ± standard deviation (SD).

After 40 d of sunflower cultivation in infested soil, the relative amount of *S. sclerotiorum* in the leaves (**Figure 8A**) and roots (**Figure 8B**) of the control treatment group was 4.85 times (*P<* 0.01, F = 432.939) and 2.68 times (*P<* 0.05, F = 77.935) higher than those in the S769-Ms treatment group, respectively. This indicated that the treatment with S769-Ms significantly reduced the content of *S. sclerotiorum* in the plants.

## 4 Discussion

### 4.1 S769 can promote sunflower seedling growth and induce resistance against *S. sclerotiorum* infection

Previous studies have demonstrated that metabolites from biocontrol microorganisms can effectively control *S*. *sclerotiorum* infection while exhibiting potential for plant growth promotion and sclerotinia disease management (Al-Quwaie, 2024). *Streptomyces* spp. species are particularly notable for their proven efficacy in both plant disease suppression and growth enhancement (Al-Quwaie, 2024). Researchers have employed diverse methods to extract bioactive components from *Streptomyces* spp., subsequently investigating their mechanisms of action and application effects (Wang et al., 2024). Current strategies for obtaining *Streptomyces* metabolites primarily involve fermentation followed by extraction of active ingredients (Ge et al., 2023; Kim et al., 2024). Tom et al. reviewed the significant advantages of *Streptomyces* spp. in promoting plant health and growth, specifically highlighting their contributions to soil disease suppression, antifungal and antibacterial activities, production of volatile compounds, and enhancement of plant biomass (Sivaprakasam et al., 2024; Viaene et al., 2016).

Empirical evidence supports the biocontrol and growth-promoting capabilities of Streptomyces spp. For instance, Salvador et al. reported a 31% increase in the fresh weight of tomato plant roots following treatment with *Streptomyces* UMAF16 (Chávez-Avila et al., 2023). Le et al. demonstrated that the fermentation filtrate of *Streptomyces* strain V31 inhibited *S. sclerotiorum* by 29.7% to 40.5%. Similarly, Dong et al. observed that *Streptomyces* NEAU-S7GS2 inhibited the mycelial growth of *S*. *sclerotiorum* by 99.1% (Liu et al., 2019). In the current study, the water extract of fermented S769 significantly inhibited the mycelial growth of *S*. *sclerotiorum*. Furthermore, Dong et al. found that *Streptomyces* NEAU-S7GS2 not only reduced soybean Sclerotinia disease incidence and disease index by 77% and 38%, respectively, but also promoted soybean growth (Liu et al., 2019). Ge et al. showed that irrigation with S769 significantly reduced watermelon Fusarium wilt incidence by 30% and increased plant biomass by 150% (Ge et al., 2024).

In the present study, treatment with S769-Ms and S769-i significantly reduced disease incidence on detached sunflower leaves. Field trials further revealed that S769 treatment substantially increased root biomass in sunflower plants, effectively decreased phytopathogen content within plant tissues, and provided significant control of pathogen damage at the seedling stage. However, during later growth stages, the disease control efficacy of S769 did not differ significantly from the control, potentially due to secondary infection by *S*. *sclerotiorum* on plants and sunflower heads, coupled with insufficient persistence of S769.

Future research should prioritize investigating the stability of the S769 compound under various storage conditions (temperature, light exposure, humidity) to determine its shelf life and mitigate potential efficacy reductions associated with short shelf life. Additionally, seed treatment and root irrigation during *S*. *sclerotiorum* infection significantly reduced the number of shriveled sunflower seeds. Therefore, reapplication of S769 during mid-to-late sunflower growth stages warrants investigation to enhance control of late *S*. *sclerotiorum* infections, thereby improving overall disease control efficiency and yield.

While this study demonstrated substantial disease control effects—including up to 65.79% inhibition of *S*. *sclerotiorum in vitro* and a 73.00% reduction in disease index in pot experiments—the economic feasibility of S769 metabolites requires further evaluation. Production costs using corn grits as the culture medium (Section 2.2) should be compared with conventional chemical treatments. In terms of scalability, the simple water extraction method described (Wu and Pang, 1979) suggests potential for large-scale production. However, commercial application would necessitate regulatory approval, contingent upon comprehensive toxicological and environmental impact assessments. Future studies should address these aspects to facilitate the practical implementation of S769 in sustainable agriculture.

### 4.2 S769 activated plant protective enzyme activities in sunflower seedlings

Laine et al. found that sunflower plants resist pathogen infection through multiple mechanisms, among which the defense enzyme system is crucial (Susi and Laine, 2015). This system includes PPO, phenylalanine ammonia-lyase (PAL), POD, and cell wall-related enzymes (Duan et al., 2024; Moosa et al., 2022). Peng et al. found that *Streptomyces* N2 stimulated fruit resistance to invading fungal pathogens, significantly reducing the reactive oxygen species content in mandarin oranges infected with mold and increasing the production of defense-related enzymes including POD, PPO, PAL, chitinase, and β-1,3-glucanase (Peng et al., 2025). In contrast, studies have shown that Trichoderma spp. can also significantly reduce the pathogenicity of *S*. *sclerotiorum* in sunflower. Specifically, treatment with fermentation filtrate of Trichoderma strain SC5 led to a significant decrease in oxalic acid (OA) content, a key virulence factor of *S*. *sclerotiorum*, with the SC5 treatment resulting in the lowest OA levels. This indicates that Trichoderma SC5 can inhibit OA production or degrade OA, thereby reducing the pathogen’s ability to cause disease (Li et al., 2024). Satyendra et al. found that in chickpeas inoculated with the pathogen *Sclerotium rolfsii*, the application of *S. griseus* significantly increased the activity of defense-related enzymes, such as PAL and PPO, and also increased the accumulation of total phenols and flavonoids. Similarly, under the same treatment, the activities of SOD, POD, and glutathione peroxidase (GPX) were significantly enhanced (Singh and Gaur, 2017). The results of this study are consistent with those of previous studies. After S769-Ms treatment, the activities of defense enzymes such as PPO, SOD, and CAT in the leaves and roots of sunflower plants grown in pots with *Sclerotinia*-infested soil were significantly higher compared to the S769-i treatment group and the control group. This indicated that after induction by S769, sunflower plants could remove the damage caused by excessive accumulation of redox reaction products in the body caused by changes in the external environment through the coordinated action of SOD, CAT, and PPO. Therefore, the findings demonstrate that S769 not only exerts direct inhibitory effects against *S*. *sclerotiorum* (as evidenced by the S769-Ms treatment group), but also confers indirect protection by inducing systemic resistance in sunflower plants. This induced resistance is mediated through the significant upregulation of key defense enzymes (PPO, SOD, CAT), enabling the plant to more effectively counteract pathogen invasion and associated oxidative stress.

### 4.3 S769 activated enzyme activities in the rhizosphere soil of sunflower seedlings

Soil enzymes mainly come from the metabolic products of soil microorganisms and the root exudates of plants (Duarte et al., 2008; Reboreda and Caçador, 2008). Enhanced rhizosphere soil enzyme activity can increase the absorption and utilization of nutrients by the roots and promote the healthy growth of plants (Hinojosa et al., 2004). Guo et al. found that inoculating *Streptomyces* Act12 into the rhizosphere soil of mustard significantly increased the activities of soil urease by 20.4% and dehydrogenase by 58.5%, while reducing the activity of alkaline phosphatase by 68.0% (Guo et al., 2019). Teslya et al. found that inoculating *Trichoderma* green wood could significantly enhance the activities of rhizosphere soil enzymes of plant seedlings, such as sucrase, acid phosphatase, and urease (Teslya et al., 2024). In this study, we found that S769-Ms treatment significantly increased the activities of sucrase, acid phosphatase, and urease in the soil, which could promote the absorption of soil nutrients by plants and the transformation process of soil nutrients, further improving the rhizosphere soil environment of sunflowers and consequently enhancing the plant’s stress resistance.

### 4.4 S769 had no significant influence on the soil microbiome of sunflower seedlings

The rhizosphere microbiome represents a distinct subset of soil microbial communities, sustained by nutrients and carbon sources derived from plant rhizodeposits. Application of biocontrol agents can significantly influence this microbiome. For instance, Zheng et al. (2024) demonstrated that soil inoculation with *Streptomyces fradiae* FJAT-31535 markedly increased the abundance of beneficial rhizosphere bacteria. Similarly, Ali et al. (2025) reported that the biocontrol strain *Bacillus subtilis* FZB42 and its metabolites effectively reshaped the microbial community structure in the plant rhizosphere soil, concurrently enhancing rice resistance to sheath blight.

However, not all biocontrol effects are mediated through microbiome modulation. Sayyed et al. (2019) found that fermentation products of *Streptomyces* strain 5406 effectively protected cotton against diverse soil-borne diseases primarily through direct antagonism against pathogens, with no significant correlation to changes in microbiome composition. Consistent with this observation, Ge et al. (2024) reported that despite S769 treatment significantly reducing watermelon fusarium wilt incidence, the relative abundance of the bacterial community showed no significant difference compared to control treatments.

In the present study, α diversity of the rhizosphere bacterial community in potted sunflowers under S769-Ms treatment was very similar to that of the control group. However, community composition analysis revealed significant differences in microbial community structure between the S769-Ms treatment and the control, aligning with the findings of Ge et al. (2024) regarding S769’s impact on composition despite unchanged overall abundance.

LEfSe analysis identified specific bacterial taxa significantly enriched in the S769-Ms-treated rhizosphere, including Chitinophagaceae, Nocardiaceae, *Flavobacterium*, and *Sphingomonas*. Notably, *Sphingomonas* has been identified as a predominant biomarker indicative of healthy rhizosphere soil (Fu et al., 2024), with *Sphingomonas* spp. established as primary contributors to the functional enrichment of this microbiome feature. Research indicates *Sphingomonas* species possess multifaceted functions, ranging from environmental contaminant remediation to plant growth promotion (Li et al., 2022). The family Chitinophagaceae is primarily associated with chitin degradation—a major component of fungal cell walls and arthropod exoskeletons—thereby contributing to nutrient cycling and microbial community stability within the soil matrix (Trinh et al., 2025). Furthermore, the genus *Flavobacterium* enhances soil fertility by decomposing complex organic matter into bioavailable forms, facilitating plant nutrient uptake and promoting plant growth (Ahmed et al., 2024).

These results suggest that S769-Ms treatment enhanced the stability of the rhizosphere microbial community structure and the robustness of network interactions. Concurrently, it effectively stimulated the enrichment of beneficial microbial groups in the soil, thereby triggering their antagonistic effects against *S*. *sclerotiorum*.

### 4.5 S769 induced disease resistance at the transcriptional level in sunflower seedlings

The root transcriptome analysis revealed that S769-Ms treatment enriched phenylpropanoid biosynthesis, cysteine and methionine metabolism, and α-linolenic acid metabolism in the plant roots. Methionine and α-linolenic acid are important precursors of ethylene and jasmonic acid (Jiang et al., 2019), while phenylpropanoid compounds play an antioxidant role in plants and help them cope with biotic and abiotic stresses, such as resisting pathogen invasion (Martinez et al., 2016). Additionally, plant hormone signal transduction, plant-pathogen interaction, and MAPK signaling pathways were upregulated by S769-Ms treatment, especially the TGACG-Binding (TGA) transcription factors pathogenesis-related protein PR-1, indole–3–acetamide synthase GH3.1, and auxin-induced protein 22D. The TGA gene family is a very important subgroup in the bZIP transcription factor family, which can bind to the as-1 region on the promoter of target genes to activate or inhibit the transcription of downstream target genes, thereby regulating plant resistance (Tian et al., 2016). The PR-1 gene is a marker of plant defense signals after pathogen attack and can be induced by salicylic acid or ethylene/jasmonic acid production (Pieterse and Van Loon, 1999). Our results were consistent with the finding by Abbasi et al. that the PR-1 gene was upregulated in susceptible tomato plants after treatment with *Streptomyces* Y28 (Abbasi et al., 2019). In the soil drench treatment, the expression levels of indole-3-acetamide synthase GH3.1 (IAA) synthesis-related genes and auxin-induced protein genes were significantly increased, which might have promoted the growth of plant roots in the later stage. These results demonstrated that the application of S769-Ms triggered plant immune responses and auxin-dependent signal transduction in the roots.

## 5 Conclusion

In conclusion, S769 could not only inhibit the phytopathogen *in vitro*, but also induced plant resistance and promoted seedling growth. Mechanistic analyses showed that S769 significantly increased the activities of antioxidant enzymes (SOD, PPO, and CAT) in plants and the activities of sucrase, acid phosphatase, and urease in soil, thereby improving the growth environment of the sunflower rhizosphere and enhancing plant stress resistance. Meanwhile, S769 application triggered plant immune responses and auxin-dependent signal transduction in the roots, thereby improving the balance between plant defense and growth. Future research should focus on optimizing application protocols, evaluating long-term stability of metabolites under field conditions, and conducting cost-benefit analyses compared to conventional chemical controls. Additionally, investigations into large-scale production methods and formulation technologies would enhance the commercial viability of S76-based biocontrol products. The integration of S769 into integrated pest management strategies could provide sustainable solutions for sunflower production while reducing reliance on chemical pesticides.

## Data Availability

The datasets presented in this study can be found in online repositories. The names of the repository/repositories and accession number(s) can be found below: https://www.ncbi.nlm.nih.gov/, PRJNA1245583 https://www.ncbi.nlm.nih.gov/, PRJNA1246416.
